# A Homozygous *MAN2B1* Missense Mutation in a Doberman Pinscher Dog with Neurodegeneration, Cytoplasmic Vacuoles, Autofluorescent Storage Granules, and an α-Mannosidase Deficiency

**DOI:** 10.3390/genes14091746

**Published:** 2023-08-31

**Authors:** Garrett Bullock, Gary S. Johnson, Savannah G. Pattridge, Tendai Mhlanga-Mutangadura, Juyuan Guo, James Cook, Rebecca S. Campbell, Charles H. Vite, Martin L. Katz

**Affiliations:** 1 Department of Veterinary Pathobiology, College of Veterinary Medicine, University of Missouri, Columbia, MO 65211, USA; gebkd2@mail.missouri.edu (G.B.); johnsongs@missouri.edu (G.S.J.); sgpgqq@missouri.edu (S.G.P.); tendai@missouri.edu (T.M.-M.); guoj@missouri.edu (J.G.); 2 Specialists in Companion Animal Neurology, Clearwater, FL 33765, USA; jrcjkcneuro@aol.com; 3 Department of Clinical Sciences and Advanced Medicine, School of Veterinary Medicine, University of Pennsylvania, Philadelphia, PA 19104, USA; rscamp@upenn.edu (R.S.C.); vite@vet.upenn.edu (C.H.V.); 4 Neurodegenerative Diseases Research Laboratory, Department of Ophthalmology, School of Medicine, University of Missouri, Columbia, MO 65212, USA

**Keywords:** lysosomal storage disease, neurodegeneration, brain, neuronal ceroid lipofuscinosis

## Abstract

A 7-month-old Doberman Pinscher dog presented with progressive neurological signs and brain atrophy suggestive of a hereditary neurodegenerative disorder. The dog was euthanized due to the progression of disease signs. Microscopic examination of tissues collected at the time of euthanasia revealed massive accumulations of vacuolar inclusions in cells throughout the central nervous system, suggestive of a lysosomal storage disorder. A whole genome sequence generated with DNA from the affected dog contained a likely causal, homozygous missense variant in *MAN2B1* that predicted an Asp104Gly amino acid substitution that was unique among whole genome sequences from over 4000 dogs. A lack of detectable α-mannosidase enzyme activity confirmed a diagnosis of a-mannosidosis. In addition to the vacuolar inclusions characteristic of α-mannosidosis, the dog exhibited accumulations of autofluorescent intracellular inclusions in some of the same tissues. The autofluorescence was similar to that which occurs in a group of lysosomal storage disorders called neuronal ceroid lipofuscinoses (NCLs). As in many of the NCLs, some of the storage bodies immunostained strongly for mitochondrial ATP synthase subunit c protein. This protein is not a substrate for α-mannosidase, so its accumulation and the development of storage body autofluorescence were likely due to a generalized impairment of lysosomal function secondary to the accumulation of α-mannosidase substrates. Thus, it appears that storage body autofluorescence and subunit c accumulation are not unique to the NCLs. Consistent with generalized lysosomal impairment, the affected dog exhibited accumulations of intracellular inclusions with varied and complex ultrastructural features characteristic of autophagolysosomes. Impaired autophagic flux may be a general feature of this class of disorders that contributes to disease pathology and could be a target for therapeutic intervention. In addition to storage body accumulation, glial activation indicative of neuroinflammation was observed in the brain and spinal cord of the proband.

## 1. Introduction

Lysosomal storage diseases (LSDs) are a diverse group of heritable disorders that share a common feature: the abnormal intra-lysosomal accumulation of one or more classes of incompletely catabolized macromolecules. Almost all of the LSDs are recessive traits. Mutations in at least 54 different genes have been identified as probable causes of human LSDs [1,2]. LSDs have been diagnosed in a variety of domestic animals, including dogs [3]. Most of the reported naturally occurring animal LSDs have been cataloged in Online Mendelian Inheritance in Animals (OMIA) [4]. One or more mutations in 20 canine orthologs of human LSD genes have been reported to be likely causes of canine LSDs. These canine genes include *ARSB*, *ATP13A2*, *CLN5*, *CLN6*, *CLN8*, *CTSD*, *GAA*, *GALC*, *GLB1*, *GUSB*, *HEXA*, *HEXB*, *IDUA*, *MANBA*, *MFSD8*, *NAGLU*, *PPT1*, *SGSH*, and *TPP1* [5,6,7,8,9,10,11,12,13,14,15,16,17,18,19,20,21,22,23,24,25,26,27,28,29]. In addition, mutations in *ARSG* and *CNP* have been reported to be likely causes of canine LSDs [30,31], although the human disorders associated with mutations in these genes have not yet been formally recognized as LSDs [32,33,34,35,36]. Conversely, there have been no previous reports of mutations in the canine orthologs of the remaining 34 documented human LSD genes, including *MAN2B1*. Biallelic mutations in *MAN2B1* have been reported to cause α-mannosidosis in human patients, cattle, cats, and Guinea pigs [37,38,39,40]. Here, we present the first reported case of canine α-mannosidosis in a Doberman Pinscher with a likely-causal homozygous *MAN2B1* missense mutation.

## 2. Materials and Methods

### 2.1. Subject Dog

**Figure 1:**
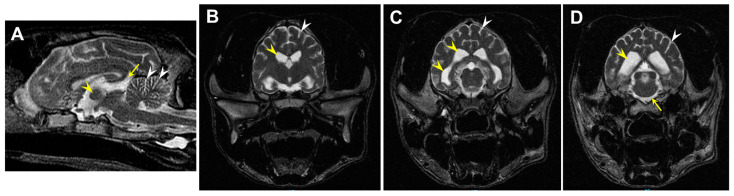
(**A**) T2-weighted mid-sagittal image demonstrating decreased size (estimated 4.8 mm) and loss of the normal round shape of the interthalamic adhesion (yellow arrowhead), increased CSF within the third ventricle at the level of the quadrigeminal cistern (yellow arrow), and between the folia of the cerebellum (white arrowheads). (**B**,**C**) T2-weighted axial images demonstrate diffuse sulcal widening (white arrowheads) and enlarged ventricles (yellow arrowheads). (**D**) The T2-weighted transverse image demonstrates diffuse sulcal widening (arrowheads), enlarged lateral ventricles (yellow arrowhead), and increased CSF surrounding the brainstem (yellow arrow).

A 7-month-old neutered male Doberman Pinscher (proband) presented for neurological evaluation by a veterinary neurologist (JC). The proband had been very unstable on its feet and had been clumsy and falling over frequently ever since he was acquired at 2 months of age by the owners. The proband had also experienced a recent episode of epistaxis. The proband had not received any medications other than routine vaccinations, deworming, and heartworm, flea, and tick prophylactics. The only abnormalities noted in a physical examination were prominent frontal sinuses and a broad calvarium not typical for the breed. Abnormalities observed upon neurological examination included slightly obtunded mentation, proprioceptive ataxia, asymmetric reduced menace response (right eye subjectively worse), and moderate divergent strabismus. No infectious agents were detected with a canine neuro PCR panel, and cell counts in the cerebrospinal fluid were within the normal range. Brain magnetic resonance imaging of the head included pre- and post-contrast T1-weighted, T2-weighted, T2 GRE, and FLAIR imaging. MRI abnormalities were consistent with brain atrophy and suggestive of a neurodegenerative disease (Figure 1). At another exam 1 month later (8 months of age), the visual responses were weaker in both eyes, and motor function had deteriorated to ambulatory paraparesis. Over the course of the disease progression, the owners reported that the proband developed aggressiveness toward other dogs, became intolerant of grooming and bathing, exhibited compulsive behaviors, including circling, and would vocalize inappropriately. The proband also became progressively uncoordinated and developed difficulty navigating stairs. Due to the progression of neurological signs, including dementia and apparent hallucinatory behavior characterized by trying to interact with non-apparent stimuli or reacting to open areas/empty spaces, the proband was euthanized at the age of 14 months. In addition to the proband, DNA samples and health information were collected on unaffected Doberman Pinschers, including the parents and one littermate of the proband.

### 2.2. Tissue Collection, Processing, and Microscopic Analyses

Following the euthanasia of the proband, the eyes were enucleated, and the central areas of the corneas were removed. One eye was then placed in a 2.5% glutaraldehyde fixative (2.5% glutaraldehyde, 0.1 M sodium cacodylate, pH 7.4), and the other eye was placed in an “immuno” fixative (3.5% formaldehyde, 0.05% glutaraldehyde, 0.12 M sodium cacodylate, 1 mM CaCl_2_, pH 7.4). The brain was removed from the skull, and slices of the parietal lobe, cerebral cortex, and cerebellar cortex were placed in immuno fixative. A second slice of each tissue was placed in EM fixative (2.0% glutaraldehyde, 1.12% formaldehyde, 0.13 M sodium cacodylate, 1 mM CaCl_2_, pH 7.4). The remainder of the brain was divided in half along the rostral–caudal midline, and one half was placed in 10% buffered formalin (Fisher Scientific, Pittsburgh, PA, USA, Cat. No. SF93-4). The other half was frozen and stored at −80 °C. Slices of the cervical spinal cord were fixed in the immuno and EM fixatives, and an additional slice was frozen and stored at −80 °C. In some hereditary neurodegenerative disorders, lysosomal storage bodies accumulate in cardiac muscle in addition to neural tissues [8]. Therefore, slices of the heart’s ventricular wall were also obtained and placed in the immuno and EM fixatives. All fixed tissues were incubated at room temperature until further processed for microscopic evaluation. Similar collection procedures were used for tissues obtained from other dogs that were used as unaffected controls.

The fixed tissue samples were prepared for light and electron microscopic evaluations and for immunohistochemistry, as described previously [31]. Slices of the immuno-fixed tissues were embedded in an OCT medium (Tissue-Tek, Sakura Finetek, Torrance, CA, USA) and cryo-sectioned at a thickness of 8 mm. The unstained sections were examined for autofluorescence as described previously [31]. Slices of both immuno-fixed and EM-fixed tissues were paraffin-embedded, and sections were either stained with hematoxylin and eosin (H&E) or immunostained for localization of glial fibrillary acidic protein (GFAP), ionized calcium-binding adapter molecule 1 (Iba1), and mitochondrial ATP synthase subunit c. Immunohistochemical staining was performed as described previously [8,31,41,42]. Slices of the EM-fixed cerebral cortex, cerebellar cortex, and cervical spinal cord were also processed for electron microscopy. This processing included secondary fixation in osmium tetroxide and embedding in epoxy resin [43]. Sections of the resin-embedded tissues were cut at a thickness of 0.2 μm, mounted on glass slides, and stained with toluidine blue. The tissue blocks were then trimmed, and sections were cut at thicknesses of 80 to 90 nm, mounted on copper grids, and stained with lead citrate and uranyl acetate. The sections mounted on glass slides were examined with a Zeiss Axiophot microscope, and the sections mounted on grids were examined with a JEOL 1400 transmission electron microscope.

### 2.3. Molecular Genetic Analysis

Genomic DNA was prepared from EDTA-anticoagulated blood as previously described [13]. DNA from the proband was submitted to the University of Missouri Genomics Technology Core Facility for library preparation and 2X150 bp paired-end sequencing on their Illumina NovaSeq 6000 sequencer. A previously described data-processing pipeline [31] was used to align the sequence reads to a current canine reference genome assembly (Dog10K_Boxer_Tasha) and to analyze them in conjunction with reads from 4034 other whole genome sequences obtained from the NCBI Sequence Read Archive (SRA). The SRA BioSample identifiers for all 4035 whole genome sequences used in this analysis are listed in Supplementary File S1.

An allelic discrimination assay was used for genotyping individual dogs for a candidate variant: a G-to-A transition at 20:43,320,989. The PCR primer sequences were 5′-CAGAATGATGTCCAGCATGCA-3′ and 5′-CTCCATCAGCAGGGACAAGAT-3′. The competing probe sequences were 5′-VIC-CAGTACATCCTAGATTCAGT-NFQ-3′ (reference allele) and 5′-FAM-CAGTACATCCTAGGTTCAGT-NFQ-3′ (variant allele).

### 2.4. Mannosidase Enzyme Activity Assays

Slices of the cerebral cortex, temporal lobe, and cerebellar cortex were dissected from frozen brain samples from the proband, from three Dachshunds with late-stage CLN2 disease [28], and from an approximately 2-year-old mixed breed hound dog with no known genetic disorder or other disease that served as a normal control. The tissues were thawed and homogenized in 154 mM NaCl and 0.2% Triton X-100 at approximately 50 mg of tissue per ml of solution, and the homogenates were frozen at −80 °C overnight. The homogenates were then centrifuged at 4 °C for 10 min at 10,000× *g*, and enzyme activities were determined in aliquots of the supernatants. The total protein contents of the samples were determined using the Bio-Rad Protein Assay Kit II with bovine serum albumin, and the absorbance was measured at 595 nm (Bio-Rad Laboratories, Hercules, CA, USA, Cat. No. 5000002). The activities of α-D-mannosidase [EC 3.2.1.24] and β-D-mannosidase [EC 3.2.1.26] were measured using 4-methylumbelliferyl α-D-mannopyranoside (GlycoSynth, Warrington, UK, Cat. No. 44081) and 4-methylumbelliferyl β-D-mannopyranoside (Sigma-Aldrich, Burlington, MA, USA, Cat. No. M0905) substrates, respectively. Enzyme activities were measured by monitoring fluorescence emission at 470 nm with excitation at 325 nm over a 30 min incubation with the reaction mixtures maintained at 37 °C. Activities are reported as pmol substrate hydrolyzed per min per mg total protein.

## 3. Results

### 3.1. Disease Pathology

MRI of the 7-month-old proband revealed marked diffuse cerebrocortical and cerebellar atrophy characterized by enlargement of the ventricles, widening of the cerebral sulci, increased space between the cerebellar folia, and atrophy of the interthalamic adhesion (Figure 1). For comparison, MR images from normal, healthy dogs can be found online by searching for “canine brain MRI atlas” using any of the common search engines.

**Figure 2:**
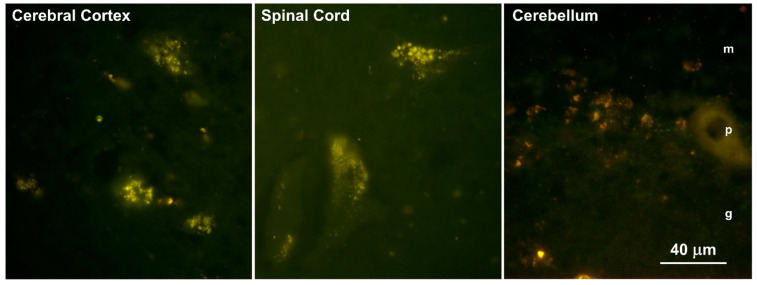
Fluorescence micrographs of unstained cryostat sections of the cerebral cortex, cervical spinal cord, and cerebellar cortex of the proband show autofluorescent inclusions in these tissues. The bar indicates the magnification of all three micrographs. The molecular (m), Purkinje cell (p), and granule cell (g) layers are indicated in the image of the cerebellum.

Fluorescence microscopic examination of unstained sections of frozen tissues obtained at the time of euthanasia revealed the presence of autofluorescent inclusions in the cerebral cortex gray matter, the cerebellar cortex, and the spinal cord (Figure 2). In the cerebral cortex, the autofluorescent inclusions were present in numerous cells distributed throughout the tissue. In the cerebellar cortex, these inclusions were present primarily in cells between the Purkinje cells but not in the Purkinje cells themselves. The autofluorescent inclusions in the spinal cord were present almost exclusively in large motor neurons. The fluorescence properties of the inclusions in all of the tissues were typical of lysosomal storage bodies that accumulate in the neuronal ceroid lipofuscinoses (emission above 510 nm with excitation at 400 to 440 nm) [9,10,21,26,27,28,31,44]. The color of the emission appeared as a golden yellow in sections of the cerebral cortex and orange in sections of the cerebellum. No inclusions with these fluorescence properties were observed in the neural retinas.

**Figure 3:**
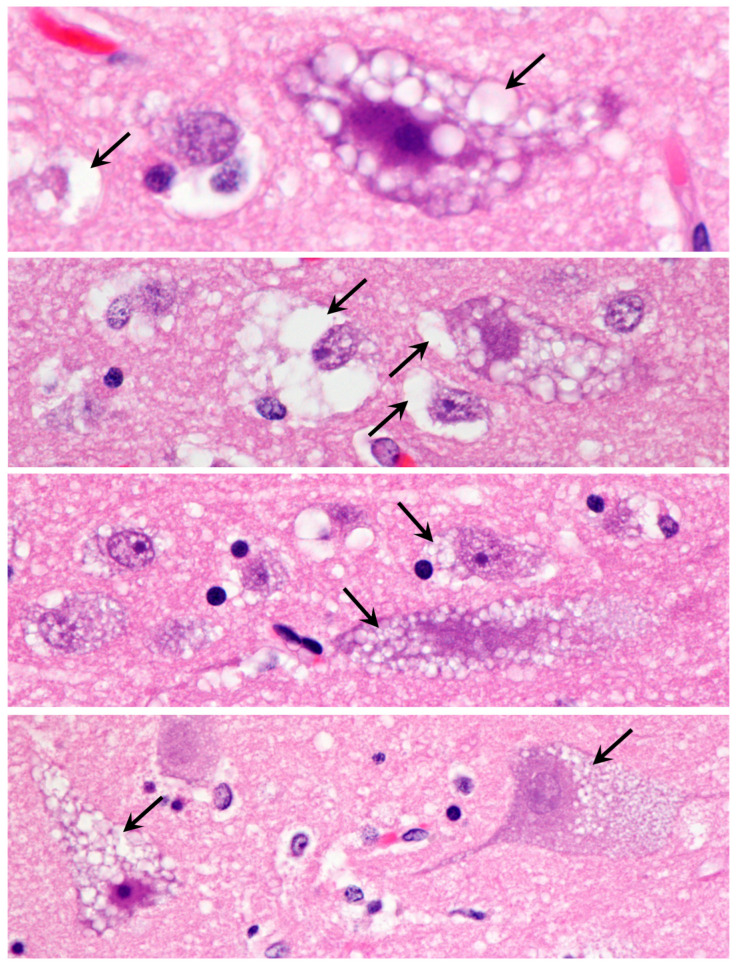
Light micrographs of cerebral cortex sections from the proband show massive accumulations of vacuolar inclusions in the vast majority of cells (arrows indicate examples of the vacuolar inclusions). H&E-stained paraffin sections.

**Figure 4:**
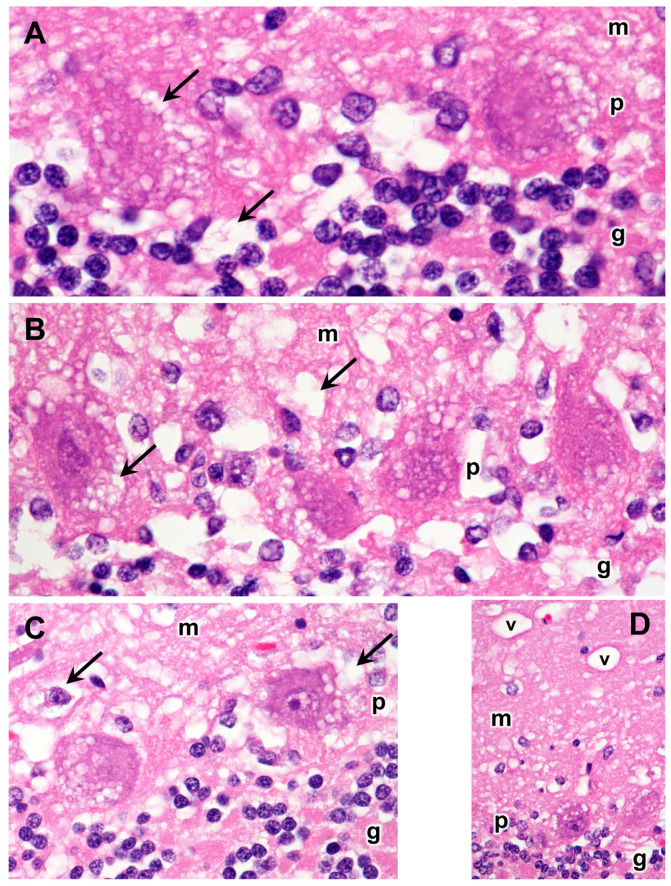
Light micrographs of cerebellar cortex sections from the proband show massive accumulations of vacuolar inclusions (examples indicated by arrows) in the vast majority of cells throughout the cortex, including the molecular (m), Purkinje (p), and granule cell (g) layers. Particularly large vacuoles (v) were present in the molecular layer. (**A**–**D**) show different regions of the cerebellar cortex. H&E-stained paraffin sections.

**Figure 5:**
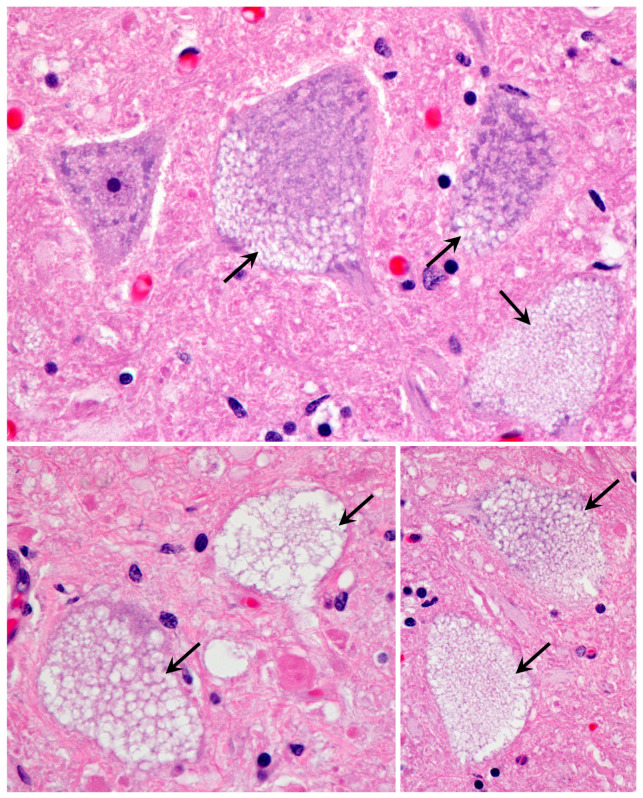
Light micrographs of cervical spinal cord sections from the proband show massive accumulations of vacuolar inclusions in large neurons (examples indicated with arrows). H&E-stained paraffin sections.

Light microscopic examination of sections from the cerebral cortex revealed that the bodies of almost all cells were nearly filled with spherical inclusions that appeared empty or stained only faintly with H&E (Figure 3). In the cerebellar cortex, the Purkinje cell bodies were mostly filled with similar spherical inclusions (Figure 4). However, in these cells, the staining within vacuoles was variable, with some being completely clear and others densely stained. The remaining cells of the cerebellar Purkinje cell layer and the molecular and granule cell layers were highly vacuolated (Figure 4). For the most part, the contents of these vacuolar inclusions did not stain with H&E. In cells of the granule cell layer, clear spherical inclusions were often packed so tightly in the cells that the boundaries between individual inclusions were barely discernible. Particularly large spherical vacuoles were present in the outer molecular layer (Figure 4D). In the cervical spinal cord, the accumulation of vacuolar inclusions was most prominent in the large motor neurons (Figure 5). In some planes of section, almost the entire cell body appeared to be filled with these inclusions.

**Figure 6:**
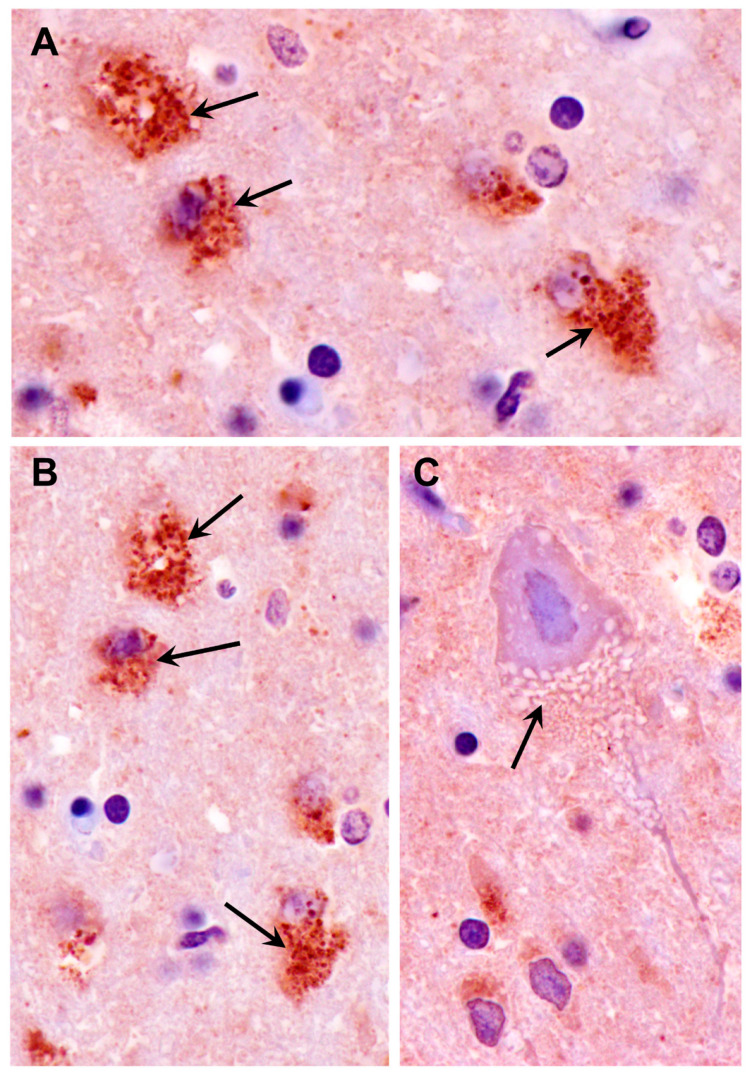
Sections of the cerebral cortex from the proband were immunostained for localization of mitochondrial ATP synthase subunit c protein. Many, but not all, cells exhibited accumulations of immunostained punctate inclusions (arrows in (**A**,**B**)). The vacuolar inclusions did not immunostain (arrow in (**C**)).

**Figure 7:**
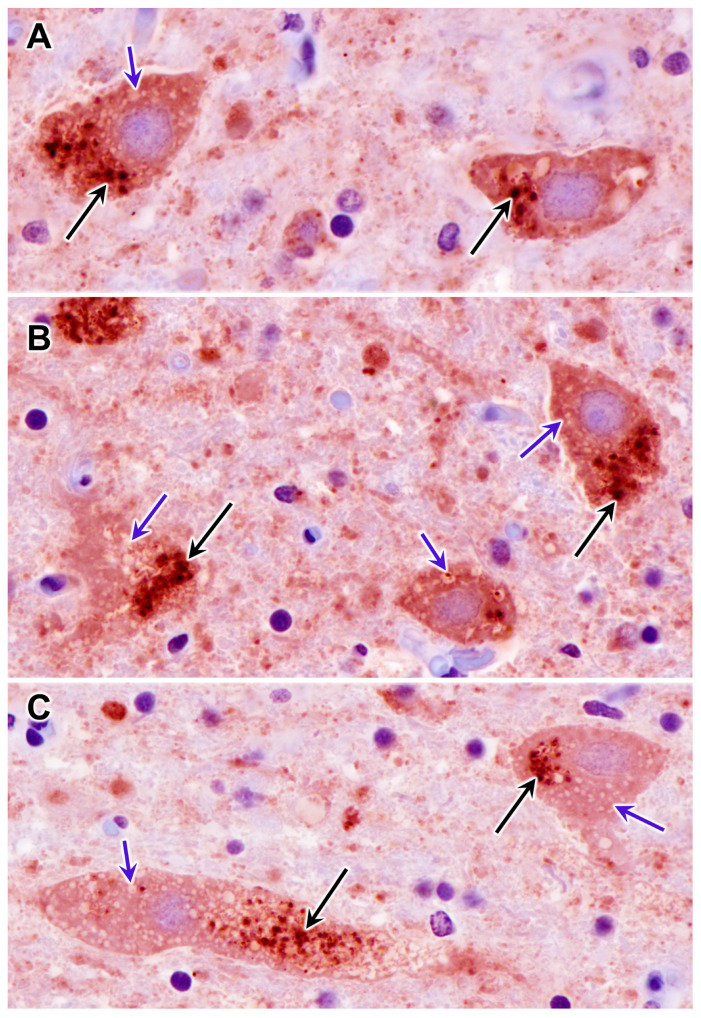
Sections of the cervical spinal cord from the proband were immunostained for localization of mitochondrial ATP synthase subunit c protein. Large neurons contained punctate inclusions that were immunopositive (black arrows) as well as unstained vacuolar inclusions (blue arrows). (**A**–**C**) show multiple neurons in the spinal cord.

**Figure 8:**
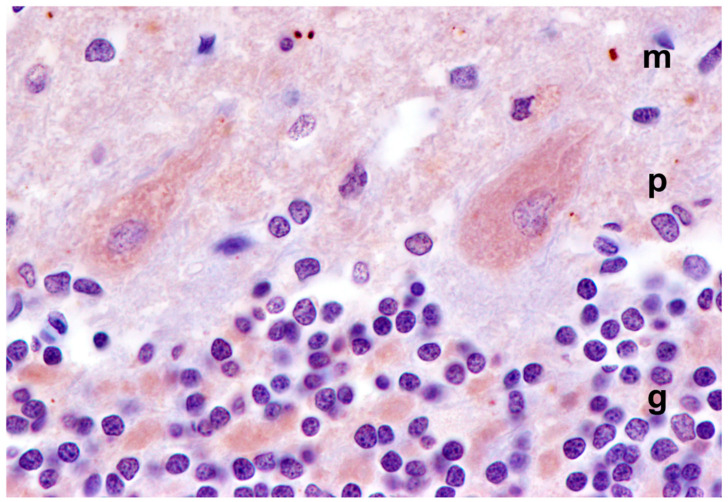
Section of the cerebellar cortex from the proband was immunostained for localization of mitochondrial ATP synthase subunit c protein. The punctate subunit immunolabel that was seen in the cerebral cortex and cervical spinal cord was not observed in the Purkinje cell layer of the cerebellar cortex (m: molecular layer; p: Purkinje cell layer; g: granule cell layer).

Because the subunit c protein of mitochondrial ATP synthase is a major component of lysosomal storage bodies and has been associated with autofluorescence in a number of lysosomal storage disorders classified as neuronal ceroid lipofuscinoses [45], sections of tissues from the proband were immunolabeled for localization of this protein. In the cerebral cortex, many cells scattered throughout the gray matter contained punctate inclusions that were labeled with the anti-subunit c antibody (Figure 6). These inclusions were distinct from the vacuolar inclusions (Figure 6C). In the cervical spinal cord, large neurons contained immunostained inclusions interspersed with unstained vacuolar inclusions (Figure 7). The subunit c immunolabeled inclusions were not observed in the cerebellar cortex, including in the cells between the Purkinje cells where the autofluorescent inclusions were observed (Figure 8).

**Figure 9:**
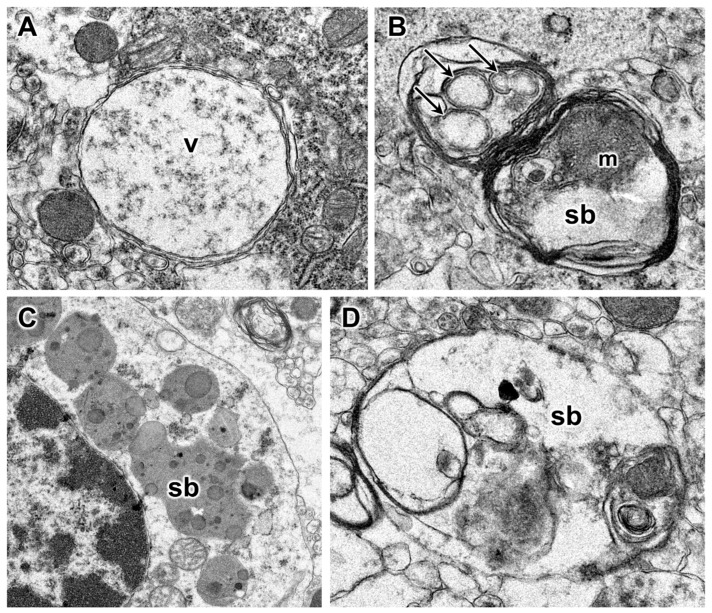
Electron micrographs illustrate the ultrastructural appearances of the vacuolar (**A**) and other types of disease-related inclusions in cerebral cortical neurons. The contents of the vacuolar inclusions (v) had a scattered flocculant appearance (**A**). The contents of other types of membrane-bounded storage bodies (sb) were heterogeneous (**B**–**D**), with the majority of the storage bodies containing membrane-like structures. Some of the inclusions within the storage bodies had double membranes characteristic of autophagosomes (arrows in **B**). Other inclusions within the storage bodies appeared to be partially degraded mitochondria (m). In many of the storage bodies, the outer membranes were multi-lamellar (**B**).

**Figure 10:**
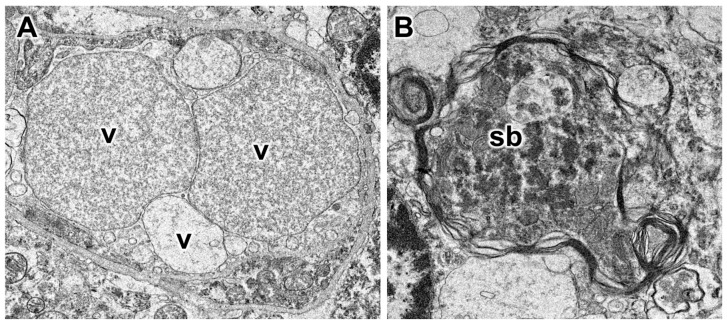
Electron micrographs illustrate the ultrastructural appearances of the vacuolar (**A**) and other types of disease-related storage bodies in cells located between the Purkinje cells of the cerebellum. The contents of the vacuolar inclusions (v) were flocculant, with variability in the density of the electron-dense material between inclusions (**A**). The contents of other disease-related storage bodies (sb) were heterogeneous in appearance. The membranes surrounding these storage bodies were often multi-lamellar (**B**).

**Figure 11:**
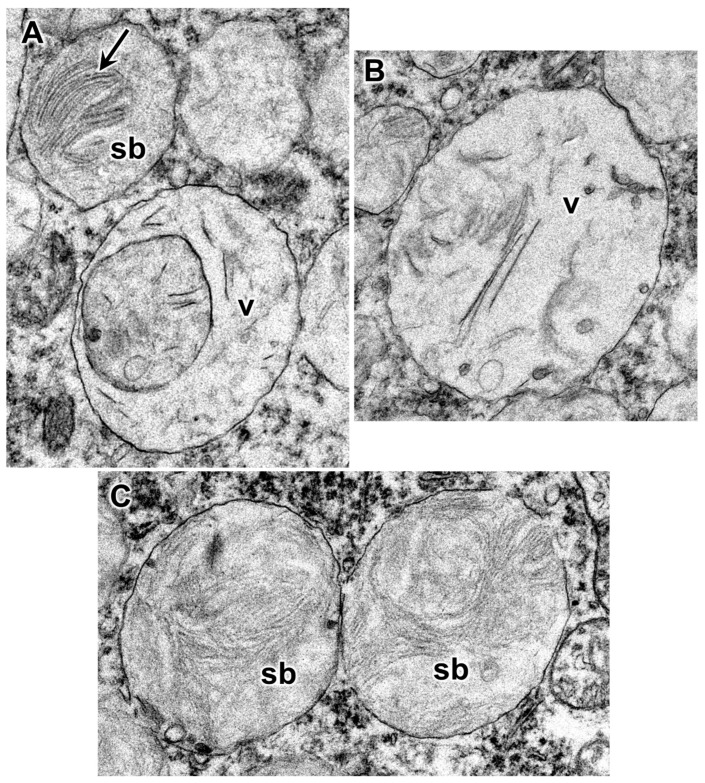
Electron micrographs show membrane-bounded inclusions in spinal cord motor neurons in the proband. (**A**–**C**) show inclusions with different-appearing contents. The contents of the majority of the inclusions (v) consisted of a relatively electron-lucent matrix in which were embedded scattered linear and vesicular structures. In some cases, an inclusion was contained within another inclusion of similar ultrastructural appearance (**A**). Some of the inclusions contained arrays of parallel multi-lamellar structures (arrow in **A**). Many of the storage bodies (sb) in these cells contained substantial amounts of membrane-like material (**C**).

**Figure 12:**
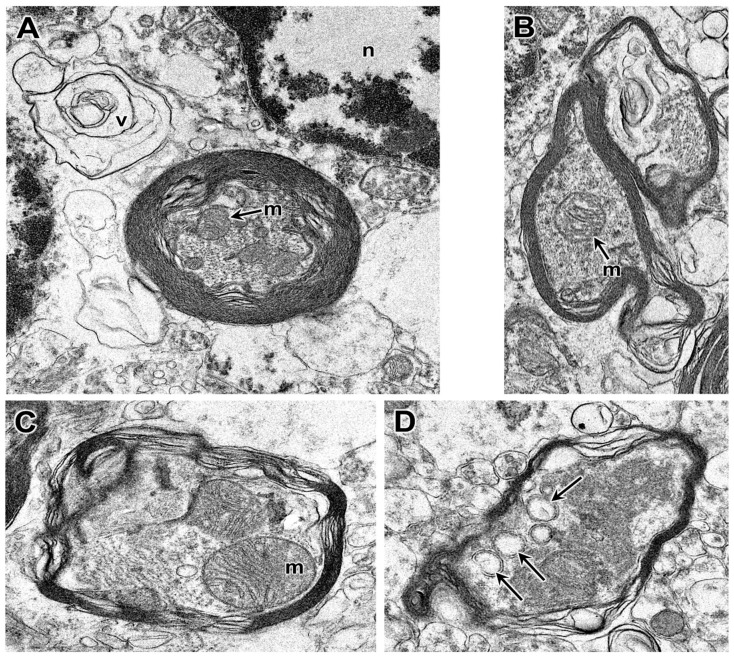
Autophagolysosome-like storage bodies in the cerebellar cortex (**A**,**B**) and cerebral cortex (**C**,**D**) from the proband dog. Some vacuolar inclusions (v) contain multiple layers of loosely packed membranes. Other storage bodies contain more tightly packed multilaminar membranes and cytoplasmic components, including intact or degrading mitochondria (m). Double-membraned vesicles were present in many of the autolysosomes (arrows in **D**). Cell nucleus: n in (**A**).

**Figure 13:**
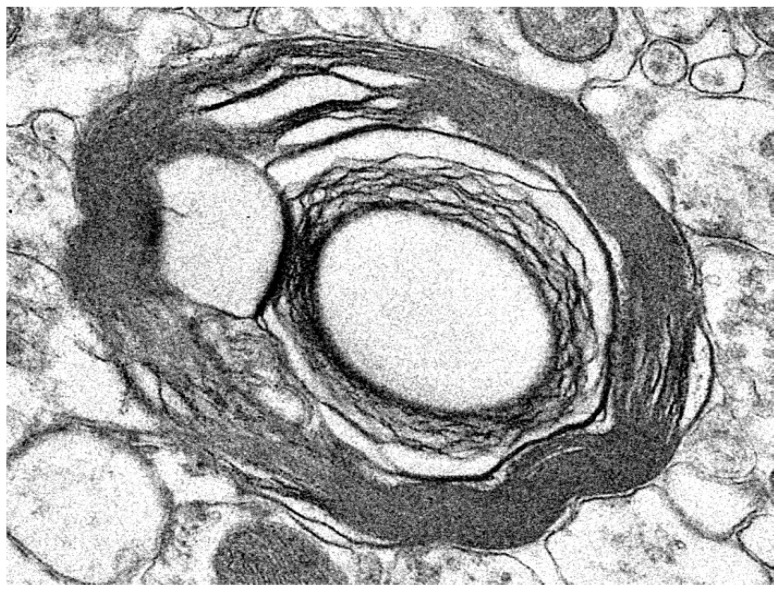
Storage body in a cerebral cortical neuron from the proband dog that consisted primarily of whorls of membrane similar to “fingerprint” profiles that are present in other lysosomal storage diseases.

Electron microscopic evaluation of the tissues from the affected dog revealed that, in addition to the vacuolar inclusions, the neurons contained a heterogeneous variety of abnormal membrane-bounded inclusions (Figure 9, Figure 10 and Figure 11). The ultrastructural appearances of some of these inclusions were characteristic of autophagolysosomes (Figure 12 and Figure 13). Autophagy is a process in which areas of the cell cytoplasm, including organelles such as mitochondria, are engulfed by a double membrane within the cell to form autophagosomes. Under normal conditions, the autophagosomes fuse with primary or secondary lysosomes to form autophagolysosomes in which the engulfed contents are degraded [46,47]. In some lysosomal storage diseases, deficiency of a specific lysosomal enzyme results in the accumulation of autophagolysosomes [48,49]. In the proband dog, numerous storage bodies in cells of the brain and spinal cord had the characteristic appearance of autophagolysosomes, containing structures that included partially degraded mitochondria and double-membraned vesicles typical of autophagosomes (Figure 9B and Figure 12). Many of the storage bodies in brain cells consisted of multi-lamellar structures surrounding engulfed cytoplasmic components (Figure 9, Figure 10 and Figure 12), and other storage bodies consisted primarily of the lamellar structures, appearing similar to the “fingerprint” profiles of storage bodies that accumulate in some other lysosomal storage diseases [48,50,51,52,53,54,55,56,57,58,59,60] (Figure 13).

**Figure 14:**
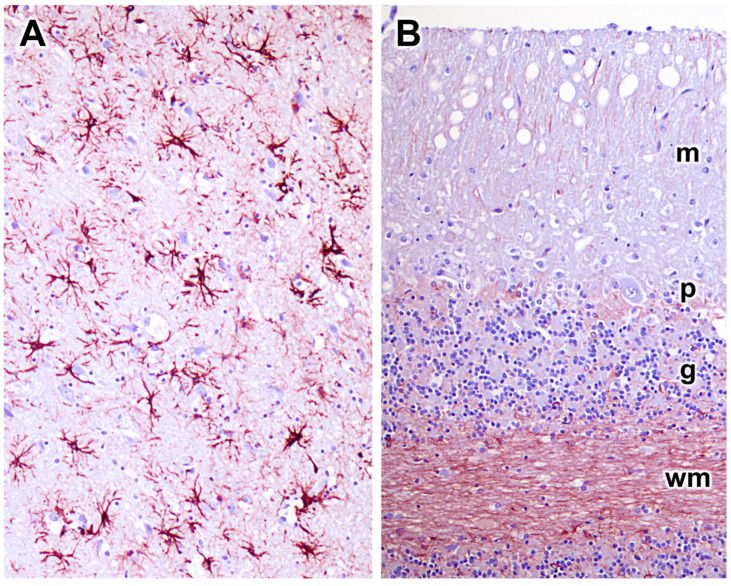
GFAP-immunostained sections of the cerebral cortex (**A**) and cerebellar cortex (**B***)* from the proband. Substantial astrogliosis was observed in many areas of the cerebral cortex but not in the cerebellar cortex. Layers of the cerebellar cortex: molecular layer (m); Purkinje cell layer (p); granule cell layer (g); and white matter (wm).

**Figure 15:**
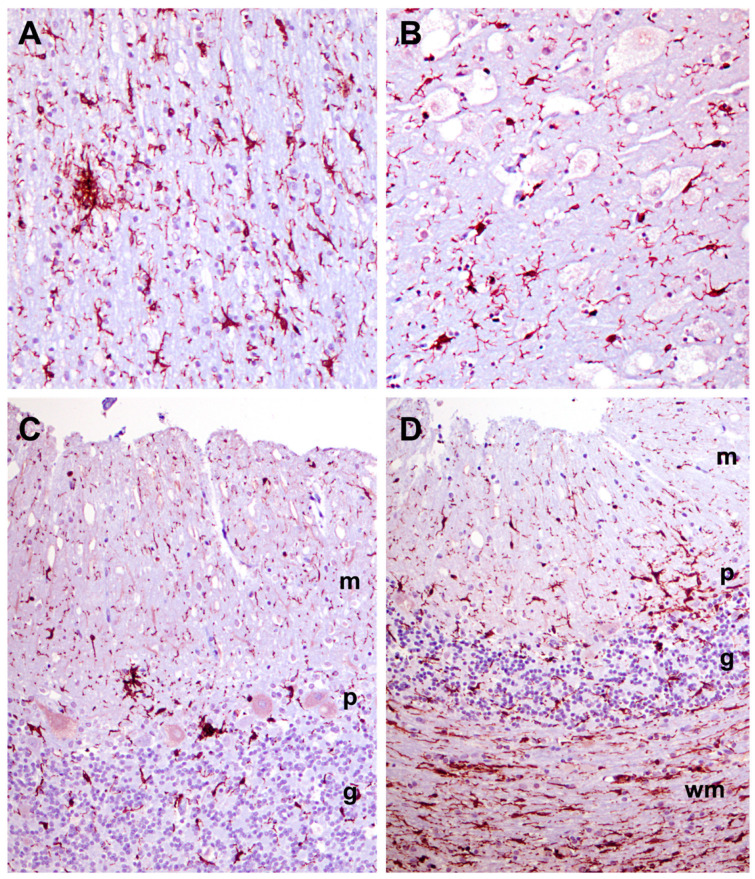
Sections of cerebral cortex (**A**,**B**) and cerebellar cortex (**C**,**D**) from the proband Iba1-immunolabeled for localization of reactive microglia. Layers of the cerebellar cortex are m: molecular layer, p: Purkinje cell layer, g: granule cell layer, and wm: white matter.

**Figure 16:**
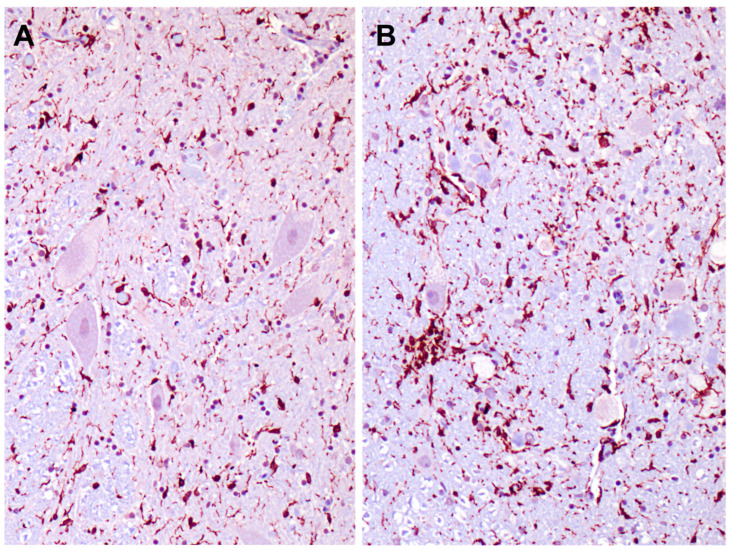
Sections of the ventral (**A**) and dorsal (**B**) horns of the cervical spinal cord from the proband Iba-1 were immunolabeled for localization of reactive microglia.

Many progressive neurodegenerative disorders are accompanied by neuroinflammation. This can be detected as elevated GFAP expression in astrocytes and Iba1 expression in microglia. GFAP immunolabeling demonstrated significant astrogliosis in the cerebral cortex gray matter (Figure 14A). Similar astrogliosis was not observed in the cerebellar cortex (Figure 14B) or in the cervical spinal cord (not shown). Reactive microglia were observed throughout the cerebral cortex and in all layers of the cerebellar cortex (Figure 15). In the cerebellar cortex, reactive microglia were particularly abundant in the white matter. Reactive microglia were also abundant throughout the spinal cord (Figure 16). 

### 3.2. Molecular Genetics

**Figure 17:**
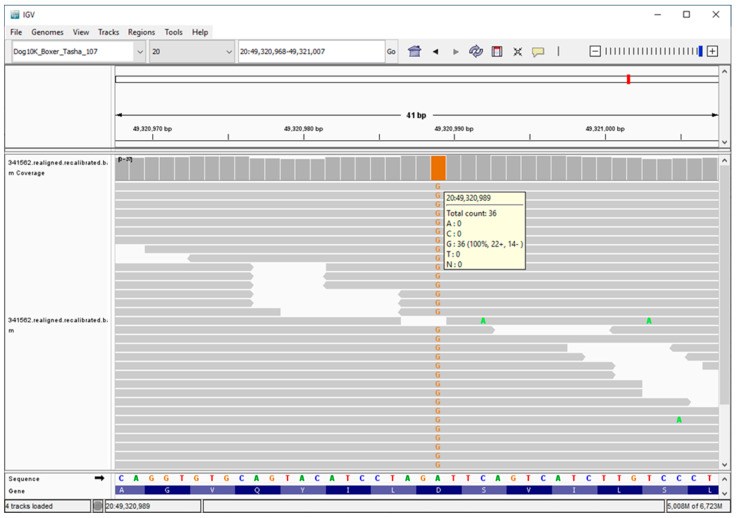
Screen shot of the proband’s whole genome sequence reads aligned to the reference sequence in the vicinity of position 49,320,898 on chromosome 20, as viewed with the Integrative Genomics Viewer (https://igv.org/app/).

We used Illumina sequencing technology and DNA from the proband to generate a 25.13-fold average coverage whole genome sequence. As indicated in the Materials and Methods section, the proband’s sequence data were analyzed in conjunction with similar data generated with DNA from 4034 dogs, including normal dogs or dogs with other diseases. These 4034 whole genome sequences served as controls and allowed us to estimate the allele frequencies of the sequence variants detected in the whole genome sequence from the proband. A total of 23,224 of the proband’s variants were predicted to alter the primary structure of the encoded protein products. This included 9578 variants in the homozygous state. These were sorted by allele frequency. The homozygous variant with the lowest allele frequency (0.000248 or 2 of 8050 called alleles) was an A-to-G transition at 3:28,472,528, which predicts a Tyr923His missense mutation in *CMYA5*, a gene that encodes a structural striated muscle component. The variant with the second lowest allele frequency (0.000249 or 2 of 8024 called alleles) was an A-to-G transition at 20:49,320,989, which predicts an Asp104Gly missense mutation in *MAN2B1*. This gene encodes lysosomal α-mannosidase and is known to harbor variants responsible for the LSD, α-mannosidosis, in a variety of species [37,38,39,40]. This variant was confirmed by inspection of the aligned sequence reads around 20:49,320,989 with the Integrative Genomics Viewer (Figure 17) [61]. Because none of the other 16 genes that harbored homozygous variants with allele frequencies <0.001 had been previously associated with LSDs, the Asp104Gly missense mutation in *MAN2B1* was considered the best candidate for causality. In support of this consideration, a blastp query indicated that an aspartate residue was conserved at a position equivalent to amino acid 104 in 100 non-canid α-mannosidase orthologs. This same query found an identical amino acid sequence from positions 95 through 107 in the 100 mammalian orthologs. In addition, PredictSNP and MutPred2, online tools for predicting how amino acid substitutions would alter protein function, both predicted that the Asp104Gly substitution would have a detrimental effect on gene function (Table 1) [62,63]. The 183 samples from Doberman Pinschers in our canine DNA archives were genotyped at the 20:49,320,989A/G variant. The proband tested homozygous for the variant G allele. His sire and dam were A/G heterozygotes, and the other 180 Doberman Pinscher samples, including one from a sibling of the proband, were A-allele homozygotes.

### 3.3. Enzymology

To validate the Asp104Gly *MAN2B1* missense mutation as the cause of the LSD in the proband, we measured the α-mannosidase enzymatic activity in the cerebral cortex and cerebellar tissues of the proband. In addition, we measured β-mannosidase activity in the same tissues to ensure that decreases in α-mannosidase enzyme activity did not result from post-mortem tissue degradation or failed recovery of lysosomal enzymes from the tissues. We also measured α- and β-mannosidase activities in brain tissues from Dachshunds with neuronal ceroid lipofuscinosis 2 (CLN2 disease), an LSD due to lysosomal tripeptidyl peptidase-1 (TPP1) deficiency [28], and from a mixed breed hound with no known disease, which served as a normal control. α-Mannosidase enzyme activity was undetectable in homogenates of the cerebral cortex and cerebellar cortex from the proband (Table 2). The limit of detection was less than 0.4% of the enzyme activities detected in these tissues in the control dog. In contrast, β-mannosidase activities were elevated in both tissues in the proband relative to the control (Table 2). Compared to the control, α-mannosidase activities were elevated in both the cerebral cortex and cerebellar cortex tissues of the TPP1-deficient Dachshunds, and β-mannosidase activities were elevated in the cerebellar cortex of the Dachshunds (Table 2).

## 4. Discussion and Conclusions

The proband was brought to the attention of a veterinary neurologist (JC) who, based on his patient’s rapidly progressing behavioral and neurological signs and MRI evidence of generalized brain atrophy, suspected that the patient had an LSD. Medical records, documentation of the owners’ observations of behavioral signs, post-mortem tissues, and EDTA-anticoagulated blood as a source of DNA were provided to the University of Missouri for diagnostic evaluation. The pattern of disease signs and progression was similar to those exhibited by dogs affected by a variety of lysosomal storage disorders [20,31,64,65]. Examination of central nervous system tissue from the proband by light and electron microscopy identified the most prominent microscopic lesion to be abundant cytoplasmic vacuole-like inclusions in many cell types. Widespread accumulation of cytoplasmic vacuolar inclusions is characteristic of specific LSDs, such as the glycoproteinoses and the mucopolysaccharidoses. These disorders accumulate water-soluble storage products that can leach out of engorged lysosomes after fixation during sample processing for microscopic examination [66]. Thus, we considered the massive accumulation of cytoplasmic vacuolar inclusions to be consistent with and supportive of the clinical impression that the proband had an LSD.

To discover a potentially causal mutation, we generated a whole genome sequence with DNA from the proband. The resulting sequence data were analyzed by identifying sequence variants that were (1) predicted to alter the primary structure of the encoded polypeptides, (2) homozygous in the proband, and (3) rare or absent in the whole genome sequences of >4000 control dogs. Eighteen genes harbored variants that met our criteria. Among these variants, an A-to-G transition at position 49,320,989 on canine chromosome 20 of the third coding exon of *MAN2B1* was considered most likely to be causal because it was the only variant that met the screening criteria and occurred within a gene previously associated with an LSD. This variant was predicted to result in an Asp104Gly amino acid substitution in lysosomal α-mannosidase, the protein encoded by *MAN2B1*. The aspartic acid at amino acid position 104 in canine α-mannosidase is highly conserved in α-mannosidase orthologs across mammalian species. Mutations in *MAN2B1*, including numerous missense mutations, have been reported to cause α-mannosidosis in a variety of species [37,38,39,67]. The lack of detectable α-mannosidase enzymatic activity in neurologic tissue from the proband was convincing confirmation that the dog had α-mannosidosis, resulting from the homozygous Asp104Gly missense mutation in *MAN2B1*. Insufficiency of specific lysosomal enzymes often results in concomitant increases in the activities of other lysosomal enzymes [28,68,69]. This may explain the higher-than-control α-mannosidase activity in the cerebrum and cerebellum from the Dachshunds with TPP1 deficiency and the higher-than-control β-mannosidase activity in the cerebrum and cerebellum from the proband and the cerebellum from the Dachshunds with TPP1 deficiency. Indeed, a number of other lysosomal enzymes are elevated in the tissues of dogs with TPP1 deficiency [28].

A human α-mannosidase-deficiency disease was first described in the late 1960s [70,71]. In 1972, a similar cattle disease was reported to result from α-mannosidase deficiency [72]. The first reports of feline α-mannosidosis cases appeared in the early 1980s [73,74,75], and the first description of α-mannosidosis in Guinea pigs was published in 1999 [76]. Surprisingly, there have been no published reports of canine α-mannosidosis until now.

Human α-mannosidosis patients typically exhibit intellectual disabilities, motor and hearing impairment, skeletal and facial abnormalities, hepatosplenomegaly, and immune deficiency with recurrent infections [77]. A severe, infantile-onset form of α-mannosidosis is characterized by rapid cognitive decline and hypotonia, with death usually occurring before the age of 10 years. The severities of clinical signs, rates of disease progression, and survival ages of human patients with later-onset α-mannosidosis have been highly variable, even among affected siblings [78]. The bases for the variability in disease signs are poorly understood [79], but in some cases, the disease severity can be correlated with the type of *MAN2B1* mutation. For example, one study reported that patients who possess at least one mutant allele that encodes a form of α-mannosidase that is successfully trafficked to the lysosomes have less severe disease phenotypes [80]. Cattle with α-mannosidosis have shown head and muscle tremors, ataxia, hypotonia, blindness, and aggression [81]. Cats with α-mannosidosis have exhibited skeletal anomalies, reduced size, ataxia, head tremor, intention tremor, strabismus, and an enlarged abdomen due to hepatomegaly [74,75]. Stunted growth, ataxia, abnormal behavior, and corneal clouding were shown in Guinea pigs with α-mannosidosis [39,76]. The proband exhibited some of these signs associated with α-mannosidosis, including intellectual disabilities, aggression, motor impairment, ataxia, and facial abnormalities.

As of 19 May 2023, 137 *MAN2B1* variant alleles were listed in the Human Gene Mutation Database as likely to have contributed to the development of human α-mannosidosis [82]. Fifty-six of these variant alleles were missense mutations. These variants result in amino acid substitutions that are distributed throughout the protein. Most of the other variant alleles were predicted to encode a truncated gene product. Single amino acid substitution variants have been shown to cause α-mannosidase deficiency for a variety of reasons, including alterations in protein folding, intracellular transport, stability, and post-translational modifications [80,83,84,85,86,87]. One of the identified *MAN2B1* missense mutations associated with α-mannosidosis involved an amino acid substitution (Asp102Asn) at the position on human *MAN2B1* that is orthologous to position p.104 on canine *MAN2B1*, the site of the likely causal homozygous missense mutation that predicts a Asp104Gly amino acid substitution in the proband.. Thus, the aspartic acid residue at this position in the protein is clearly essential for normal α-mannosidase function.

The predominant microscopic lesions in tissues from the proband were cytoplasmic vacuolar inclusions, which were numerous and found in many cell types. Similar lesions have been reported from human patients with α-mannosidosis and from cases of α-mannosidosis in cattle, cats, and Guinea pigs [88,89,90,91,92]. These vacuoles form because α-mannosidase deficiency blocks essential steps in the lysosomal catabolism of the asparagine-linked carbohydrate side chains of glycoproteins, resulting in the accumulation of incompletely digested, mannose-rich, water-soluble oligosaccharides [93]. 

In addition to the abundant cytoplasmic vacuolar inclusions, we detected less numerous abnormal storage granules with distinct characteristics, including lipofuscin-like autofluorescence, accumulation of mitochondrial ATP synthase subunit c protein, and complex heterogeneous ultrastructures. Comparisons of the tissue-specific and subcellular distributions of the vacuolar inclusions with the distributions of the autofluorescent puncta and the subunit c immunostaining intracellular inclusions indicated that most of the vacuoles are not autofluorescent and do not accumulate subunit c. It seems likely that at least some of the storage granules with the nonvacuolar ultrastructures are the source of the subunit c immunostaining and the autofluorescence. A well-established association between ATP synthase subunit c accumulation and autofluorescence in storage granules from a variety of neuronal ceroid lipofuscinoses may account for autofluorescence in the cerebrum and spinal cord [94]. However, autofluorescence in some cells of the cerebellum was not accompanied by detectable subunit c immunostaining, suggesting the molecular composition of the storage material was different in the cerebellar cells than that in the cerebral cortex and spinal cord. Consistent with this possibility, the color of the fluorescence emission of the storage material was similar in sections of the cerebral cortex and spinal cord but shifted red in sections of the cerebellum. Similar variations between tissues in fluorescence emission color, as well as in storage body ultrastructure, are seen in the neuronal ceroid lipofuscinoses and similar lysosomal storage disorders [6,8,9,12,13,14,15,22,27,28,31,42,64,95,96,97,98,99]. For example, in dogs with the form of neuronal ceroid lipofuscinosis resulting from a mutation in *ATP13A2*, the fluorescence emission color of storage bodies ranged from greenish–yellow to golden–yellow to orange–yellow in cells of different tissues, and there were tissue-specific differences in storage body ultrastructure [6].

We have not found previous reports of autofluorescent cytoplasmic inclusions or ATP synthase subunit c accumulation in tissues from cases of α-mannosidosis in any species. Nonvacuolar inclusions with an ultrastructure similar to some of the inclusions from the proband have been reported to occur in certain cerebral neurons from cats with α-mannosidosis and attributed to secondary lipid accumulation [100,101]. The term secondary accumulation refers to the LSD-related lysosomal accumulation of molecular components of cells that bear no direct relation to the metabolic pathway altered by the primary genetic defect [101,102,103,104]. In the proband, some of the disease-related inclusions appeared to be autophagosomes containing intact or partially degraded mitochondria. Failure to degrade the subunit c protein from these mitochondria could account for the accumulation of this protein in storage bodies. Subunit c is a small, very hydrophobic protein that tends to aggregate [105]. Aggregation of this protein may inhibit its degradation and contribute to the formation of membrane-like structures within the storage bodies. Several LSDs have been associated with impaired autophagic flux and the accumulation of autophagosomes/autophagolysosomes [104,106,107]. Inclusions with ultrastructures similar to some of the proband’s nonvacuolar inclusions have been shown in brain cells from murine LSD models and classified as autophagosomes [48,108,109,110]. Although we have not found previous reports of impaired autophagic flux in α-mannosidosis, impaired autophagic flux has been demonstrated in a mouse model of the LSD, fucosidosis [111]. Similar to α-mannosidosis, fucosidosis is associated with the lysosomal accumulation of oligosaccharides due to a deficiency of a gene that encodes a lysosomal glycosidase required for the complete catabolism of the asparagine-linked glycan side chains of glycoproteins [112]. Thus, it is plausible that some of the proband’s nonvacuolar inclusions represent secondary lipid accumulation and/or that some of these inclusions are autophagosomes that accumulated due to impaired autophagic flux. It is not unexpected that the molecular composition of the secondary autophagolysosome-like inclusions would vary between tissues and cell types since the molecular makeup of specialized cells is quite distinct. This heterogeneity is reflected in the variability of storage body ultrastructure among different cells in the LSDs.

Lipofuscin-like autofluorescence and subunit c immunoreactivity of lysosomal storage bodies are characteristic of a class of LSDs categorized as neuronal ceroid lipofuscinoses (NCLs). The accumulation of storage bodies with these features has been used as a basis for classifying disorders as NCLs. The case described in this study indicates that the accumulation of storage bodies with these features is not exclusive to the NCLs. However, in contrast to the recognized NCLs, in which most if not all of the storage bodies exhibit lipofuscin-like autofluorescence and, in most forms of NCL, subunit c protein accumulation as well, only a minority of the storage bodies had these features in the proband. The accumulation of the latter type of storage bodies likely reflects an effect of the accumulation of α-mannosidase substrates on general lysosomal function, leading to the accumulation of compounds that are not directly related to α-mannosidase enzymatic activity. In the proband, these secondary storage bodies had ultrastructures characteristic of autophagolysosomes. Some of the storage bodies in the affected dog had ultrastructural features intermediate between the abundant vacuolar inclusions and the less abundant autophagolysosome-like inclusions. The presence of these intermediate inclusions is consistent with secondary accumulation of materials that are not α-mannosidase substrates. 

In summary, a dog that exhibited a progressive neurological disease with global brain atrophy was found to harbor a homozygous missense variant in *MAN2B1*. Tissues from the affected dog lacked detectable α-mannosidase enzymatic activity, and the dog exhibited massive accumulations of vacuolar inclusions in most cells of the brain, consistent with features of α-mannosidosis in other species. In addition to the more numerous vacuolar inclusions, the disease was accompanied by an apparently secondary accumulation of storage bodies with heterogeneous, complex ultrastructure, including some that exhibited lipofuscin-like autofluorescence and/or accumulated mitochondrial ATP synthase subunit c protein. This suggests that a lack of α-mannosidase resulted in general impairment of the autophagolysosomal system. A number of studies suggest that treatments that alter the function of the autophagolysosomal system may have therapeutic benefits in treating lysosomal storage diseases like α-mannosidosis [113,114,115,116,117].

## Figures and Tables

**Table 1:** Results of in silico protein prediction tools for the pathogenicity of canine *MAN2B1*:p.Asp104Gly.

Prediction Tool	Variant Score/Score for Deleterious Prediction	Prediction of Pathogenicity
PredictSNP *	87%	Deleterious *
MutPred2	0.717	Likely pathogenic

* Each of the individual programs in the PredictSNP suite classified the p.Asp104Gly missense variant as deleterious.

**Table 2:** Mannosidase Enzyme Activities in Dog Tissues.

Enzyme	Dog	Tissue	Activity *
α-mannosidase	Proband	Cerebral Cortex	0
α-mannosidase	CLN2 Dachshund (n = 3)	Cerebral Cortex	137 ^†^
α-mannosidase	Normal Control	Cerebral Cortex	89
α-mannosidase	Proband	Cerebellum	0
α-mannosidase	CLN2 Dachshund (n = 3)	Cerebellum	141 ^†^
α-mannosidase	Normal Control	Cerebellum	59
β-mannosidase	Proband	Cerebral Cortex	147
β-mannosidase	CLN2 Dachshund (n = 3)	Cerebral Cortex	71 ^†^
β-mannosidase	Normal Control	Cerebral Cortex	90
β-mannosidase	Proband	Cerebellum	152
β-mannosidase	CLN2 Dachshund (n = 3)	Cerebellum	111 ^†^
β-mannosidase	Normal Control	Cerebellum	65

* pmol substrate hydrolyzed per min per mg total protein; limit of quantitation: 0.5 pmol substrate hydrolyzed/min/mg protein. ^†^ Mean of the separately determined activities from three individual TPP1-deficient Dachshunds.

## Data Availability

DNA sequence data for the proband have been archived and deposited in the NCBI Sequence Read Archive as BioSample SAMN24256750.

## Supplementary Materials

The following are available online at https://www.mdpi.com/article/10.3390/genes14091746/s1; Supplementary File S1 consists of a list of the NCBI Sequence Read Archive BioSample identifiers for the whole genome sequences compared in this study.
